# CRISPR-Cas systems: Overview, innovations and applications in human disease research and gene therapy

**DOI:** 10.1016/j.csbj.2020.08.031

**Published:** 2020-09-08

**Authors:** Yuanyuan Xu, Zhanjun Li

**Affiliations:** Key Laboratory of Zoonosis Research, Ministry of Education, College of Animal Science, Jilin University, Changchun 130062, China

**Keywords:** CRISPR, Cas9, Genome editing, Human disease models, Rabbit, Gene therapy, Off target effects

## Abstract

Genome editing is the modification of genomic DNA at a specific target site in a wide variety of cell types and organisms, including insertion, deletion and replacement of DNA, resulting in inactivation of target genes, acquisition of novel genetic traits and correction of pathogenic gene mutations. Due to the advantages of simple design, low cost, high efficiency, good repeatability and short-cycle, CRISPR-Cas systems have become the most widely used genome editing technology in molecular biology laboratories all around the world. In this review, an overview of the CRISPR-Cas systems will be introduced, including the innovations, the applications in human disease research and gene therapy, as well as the challenges and opportunities that will be faced in the practical application of CRISPR-Cas systems.

## 1 Introduction

Genome editing is the modification of genomic DNA at a specific target site in a wide variety of cell types and organisms, including insertion, deletion and replacement of DNA, resulting in inactivation of target genes, acquisition of novel genetic traits and correction of pathogenic gene mutations [1], [2], [3]. In recent years, with the rapid development of life sciences, genome editing technology has become the most efficient method to study gene function, explore the pathogenesis of hereditary diseases, develop novel targets for gene therapy, breed crop varieties, and so on [4], [5], [6], [7].

At present, there are three mainstream genome editing tools in the world, zinc finger nucleases (ZFNs), transcription activator-like effector nucleases (TALENs) and the RNA-guided CRISPR (clustered regularly interspaced short palindromic repeats)-Cas (CRISPR-associated) nucleases systems [8], [9], [10]. Due to the advantages of simple design, low cost, high efficiency, good repeatability and short-cycle, CRISPR-Cas systems have become the most widely used genome editing technology in molecular biology laboratories all around the world [11], [12]. In this review, an overview of the CRISPR-Cas systems will be introduced, including the innovations and applications in human disease research and gene therapy, as well as the challenges and opportunities that will be faced in the practical application of CRISPR-Cas systems.

## 2 Overview of CRISPR-Cas systems

CRISPR-Cas is an adaptive immune system existing in most bacteria and archaea, preventing them from being infected by phages, viruses and other foreign genetic elements [13], [14]. It is composed of CRISPR repeat-spacer arrays, which can be further transcribed into CRISPR RNA (crRNA) and *trans*-activating CRISPR RNA (tracrRNA), and a set of CRISPR-associated (cas) genes which encode Cas proteins with endonuclease activity [15]. When the prokaryotes are invaded by foreign genetic elements, the foreign DNA can be cut into short fragments by Cas proteins, then the DNA fragments will be integrated into the CRISPR array as new spacers [16]. Once the same invader invades again, crRNA will quickly recognize and pair with the foreign DNA, which guides Cas protein to cleave target sequences of foreign DNA, thereby protecting the host [16].

CRISPR-Cas systems can be classified into 2 classes (Class 1 and Class 2), 6 types (I to VI) and several subtypes, with multi-Cas protein effector complexes in Class 1 systems (Type I, III, and IV) and a single effector protein in Class 2 systems (Type II, V, and VI) [17], [18]. The classification, representative members, and typical characteristics of each CRISPR-Cas system are summarized in Table 1 [10], [12], [15], [16], [17], [18].

**Table 1:** Summary of CRISPR-Cas systems.

Class	Type	Subtype	Effector	Target	Nuclease domains	TracrRNA requirement	PAM/PFS
1 (multi-Cas proteins)	Ⅰ	A, B, C, D, E, F, U	Cascade	dsDNA	HD fused to Cas3	No	–
1	III	A, B, C, D	Cascade	ssRNA	HD fused to Cas10	No	–
1	Ⅳ	A, B	Cascade	dsDNA	unknown	No	–
2 (single-Cas protein)	Ⅱ	A	SpCas9	dsDNA	RuvC, HNH	Yes	NGG
2	Ⅱ	A	SaCas9	dsDNA	RuvC, HNH	Yes	NNGRRT
2	Ⅱ	B	FnCas9	dsDNA/ssRNA	RuvC, HNH	Yes	NGG
2	Ⅱ	C	NmCas9	dsDNA	RuvC, HNH	Yes	NNNNGATT
2	Ⅴ	A	Cas12a (Cpf1)	dsDNA	RuvC, Nuc	No	5′ AT-rich PAM
2	Ⅴ	B	Cas12b (C2c1)	dsDNA	RuvC	Yes	5′ AT-rich PAM
2	Ⅴ	C	Cas12c (C2c3)	dsDNA	RuvC	Yes	5′ AT-rich PAM
2	VI	A	Cas13a (C2c2)	ssRNA	2xHEPN	No	3′PFS: non-G
2	VI	B	Cas13b (C2c4)	ssRNA	2xHEPN	No	5′PFS: non-C; 3′PFS:NAN/NNA
2	VI	C	Cas13c (C2c7)	ssRNA	2xHEPN	No	–
2	VI	D	Cas13d	ssRNA	2xHEPN	No	–

Type II CRISPR-Cas9 system derived from *Streptococcus pyogene*s (SpCas9) is one of the best characterized and most commonly used category in numerous CRISPR-Cas systems [18], [19]. The main components of CRISPR-Cas9 system are RNA-guided Cas9 endonuclease and a single-guide RNA (sgRNA) [20]. The Cas9 protein possesses two nuclease domains, named HNH and RuvC, and each cleaves one strand of the target double-stranded DNA [21]. A single-guide RNA (sgRNA) is a simplified combination of crRNA and tracrRNA [22]. The Cas9 nuclease and sgRNA form a Cas9 ribonucleoprotein (RNP), which can bind and cleave the specific DNA target [23]. Furthermore, a protospacer adjacent motif (PAM) sequence is required for Cas9 protein’s binding to the target DNA [20].

**Fig. 1:**
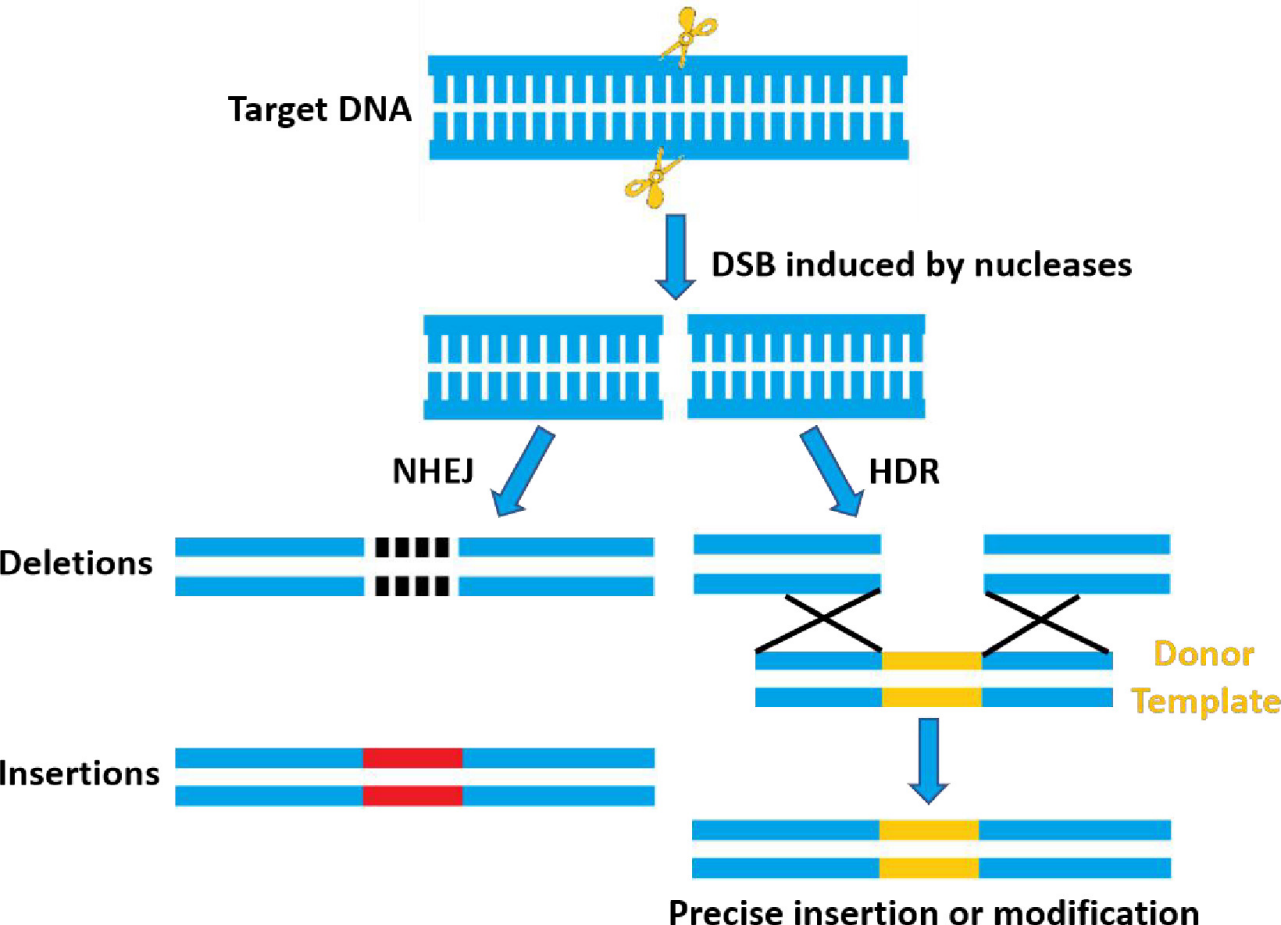
Mechanism of genome editing. Double-strand break (DSB) induced by nucleases can be repaired by non-homologous end joining (NHEJ) or homology-directed repair (HDR) pathways. NHEJ can introduce random insertions or deletions (indels) of varying length at the site of the DSB. Alternatively, HDR can introduce precise genomic modifications at the target site by using a homologous DNA donor template.

During genome editing process, sgRNA recruits Cas9 endonuclease to a specific site in the genome to generate a double-stranded break (DSB), which can be repaired by two endogenous self-repair mechanisms, the error-prone non-homologous end joining (NHEJ) pathway or the homology-directed repair (HDR) pathway [24]. Under most conditions, NHEJ is more efficient than HDR, for it is active in about 90% of the cell cycle and not dependent on nearby homology donor [25]. NHEJ can introduce random insertions or deletions (indels) into the cleavage sites, leading to the generation of frameshift mutations or premature stop codons within the open reading frame (ORF) of the target genes, finally inactivating the target genes [26], [27]. Alternatively, HDR can introduce precise genomic modifications at the target site by using a homologous DNA repair template [28], [29] (Fig. 1). Furthermore, large fragment deletions and simultaneous knockout of multiple genes could be achieved by using multiple sgRNAs targeting one single gene or more [30], [31].

## 3 Innovations of CRISPR-Cas systems

CRISPR-Cas systems have become the most favorite genome editing tool in the molecular biology laboratory since they were confirmed to have genome editing capabilities in 2012 [23]. They have made numerous achievements in the field of correcting pathogenic mutations, searching for essential genes for cancer immunotherapy, and solving key problems in organ xenotransplantation [5], [32], [33]. Unfortunately, there are still some limitations which need to solve in CRISPR-Cas systems, such as potential off-target effects, limited genome-targeting scope restricted by PAM sequences, and low efficiency and specificity [34], [35]. Therefore, many research teams have been trying to improve this tool.

### 3.1 Dead-Cas9 system

By introducing two point mutations, H840A and D10A, into HNH and RuvC nuclease domain, researchers have obtained a nuclease dead Cas9 (dCas9) [36]. The dCas9 lacks DNA cleavage activity, but DNA binding activity is not affected. Then, by fusing transcriptional activators or repressors to dCas9, the CRISPR-dCas9 system can be used to activate (CRISPRa) or inhibit (CRISPRi) transcription of target genes [37], [38]. Additionally, dCas9 can be fused to various effector domains, which enables sequence-specific recruitment of fluorescent proteins for genome imaging and epigenetic modifiers for epigenetic modification [39], [40]. Furthermore, this system is easy to operate and allows simultaneous manipulation of multiple genes within a cell [38].

### 3.2 Base editing system

In order to improve the efficiency of site-directed mutagenesis, base editing systems containing dCas9 coupled with cytosine deaminase (cytidine base editor, CBE) or adenosine deaminase (adenine base editor, ABE) have been developed [41], [42]. It can introduce C·G to T·A or A·T to G·C point mutations into the editing window of the sgRNA target sites without double-stranded DNA cleavage [41], [42]. Since base editing systems avoid the generation of random insertions or deletions to a great extent, the results of gene mutation are more predictive. However, owing to the restriction of base editing window, base editing systems are not suitable for any target sequence in the genome. Accordingly, C-rich sequences, for example, would produce a lot of off-target mutations [43]. Therefore, researchers have always been trying to develop and optimize novel base editing systems to overcome this drawback [44]. At present, base editing systems have been widely used in various cell lines, human embryos, bacteria, plants and animals for efficient site-directed mutagenesis, which may have broad application prospects in basic research, biotechnology and gene therapy [45], [46], [47]. In theory, 3956 gene variants existing in Clin var database could be repaired by base substitution of C-T or G-A [42], [48].

### 3.3 Cas9 variant system

An NGG PAM at the 3′ end of the target DNA site is essential for the recognization and cleavage of the target gene by Cas9 protein [20]. Besides classical NGG PAM sites, other PAM sites such as NGA and NAG also exist, but their efficiency of genome editing is not high [49]. However, such PAM sites only exist in about one-sixteenth of the human genome, thereby largely restricting the targetable genomic loci. For this purpose, several Cas9 variants have been developed to expand PAM compatibility.

In 2018, David Liu *et al.* [50] developed xCas9 by phage-assisted continuous evolution (PACE), which can recognize multiple PAMs (NG, GAA, GAT, etc.). In the latter half of the same year, Nishimasu *et al.* developed SpCas9-NG, which can recognize relaxed NG PAMs [51]. In 2020, Miller *et al.* developed three new SpCas9 variants recognizing non-G PAMs, such as NRRH, NRCH and NRTH PAMs [52]. Later in the same year, Walton *et al.* developed a SpCas9 variant named SpG, which is capable of targeting an expanded set of NGN PAMs [53]. Subsequently, they optimized the SpG system and developed a near-PAMless variant named SpRY, which is capable of editing nearly all PAMs (NRN and NYN PAMs) [53].

By using these Cas9 variants, researchers have repaired some previously inaccessible disease-relevant genetic variants [51], [52], [53]. However, there are still some drawbacks in these variants, such as low efficiency and cleavage activity [50], [51]. Therefore, they should be further improved by molecular engineering in order to expand the applications of SpCas9 in disease-relevant genome editing.

### 3.4 RNA editing system

In addition to editing DNA, CRISPR-Cas systems can also edit RNA. Class 2 Type VI CRISPR-Cas13 systems contain a single RNA-guided Cas13 protein with ribonuclease activity, which can bind to target single-stranded RNA (ssRNA) and specifically cleave the target [54]. To date, four Cas13 proteins have been identified: Cas13a (also known as C2c2), Cas13b, Cas13c and Cas13d [55]. They have successfully been applied in RNA knockdown, transcript labeling, splicing regulation and virus detection [56], [57], [58]. Later, Feng Zhang *et al.* developed two RNA base edting systems (REPAIR system, enables A-to-I (G) replacement; RESCUE system, enables C-to-U replacement) by fusing catalytically inactivated Cas13 (dCas13) with the adenine/cytidine deaminase domain of ADAR2 (adenosine deaminase acting on RNA type 2) [59], [60].

Compared with DNA editing, RNA editing has the advantages of high efficiency and high specificity. Furthermore, it can make temporary, reversible genetic edits to the genome, avoiding the potential risks and ethical issues caused by permanent genome editing [61], [62]. At present, RNA editing has been widely used for pre-clinical studies of various diseases, which opens a new era for RNA level research, diagnosis and treatment.

### 3.5 Prime editing system

Recently, Anzalone *et al.* developed a novel genome editing technology, named prime editing, which can mediate targeted insertions, deletions and all 12 types of base substitutions without double-strand breaks or donor DNA templates [63]. This system contains a catalytically impaired Cas9 fused to a reverse transcriptase and a prime editing guide RNA (pegRNA) with functions of specifying the target site and encoding the desired edit [63]. After Cas9 cleaves the target site, the reverse transcriptase uses pegRNA as a template for reverse transcription, and then, new genetic information can be written into the target site [63]. Prime editing can effectively improve the efficiency and accuracy of genome editing, and significantly expand the scope of genome editing in biological and therapeutic research. In theory, it is possible to correct up to 89% known disease-causing gene mutations [63]. Nevertheless, as a novel genome editing technique, more research is still needed to further understand and improve prime editing system.

## 4 Applications of CRISPR-Cas systems in human disease research

### 4.1 Applications of CRISPR-Cas systems in establishing animal and cell models of human diseases

So far, as a rapid and efficient genome editing tool, CRISPR-Cas systems have been extensively used in a variety of species, including bacteria, yeast, tobacco, Arabidopsis, sorghum, rice, Caenorhabditis elegans, Drosophila, zebrafish, Xenopus laevis, mouse, rat, rabbit, dog, sheep, pig and monkey [64], [65], [66], [67], [68], [69], [70], [71], [72], [73], [74], [75], [76], [77], [78], as well as various human cell lines, such as tumor cells, adult cells and stem cells [79], [80]. In medical field, the most important application of CRISPR-Cas systems is to establish genetically modified animal and cell models of many human diseases, including gene knockout models, exogenous gene knock-in models, and site directed mutagenesis models [80], [81].

(1) Establishing animal models of human diseases
  Animal models are crucial tools for understanding gene function, exploring pathogenesis of human diseases and developing new drugs. However, traditional methods for generating animal models are complex, costly and time-consuming, which severely limit the application of animal models in basic medical research and preclinical studies [82]. Since the discovery of CRISPR-Cas systems, a series of genetically modified animal models have successfully been generated in a highly efficient manner [72], [73], [74], [75], [76], [77], [78].

Among numerous model animals, mice are widely used for scientific studies and recognized as the most important model animals in human disease research [83]. So far, researchers have successfully generated many genetically modified mouse models, such as cancer, cardiovascular disease, cardiomyopathy, Huntington's disease, albino, deafness, hemophilia B, obesity, urea cycle disorder and muscular dystrophy [84], [85], [86], [87], [88], [89], [90], [91], [92], [93]. Nevertheless, owing to the great species differences between humans and rodents, they can’t provide effective assessment and long-term follow-up for research and treatment of human diseases [94]. Therefore, the application of larger model animals, such as rabbits, pigs and non-human primates, is becoming more and more widespread [74], [77], [78]. With the development of CRISPR-Cas systems, generating larger animal models for human diseases has become a reality, which greatly enriches the disease model resource bank.

**Fig. 2:**
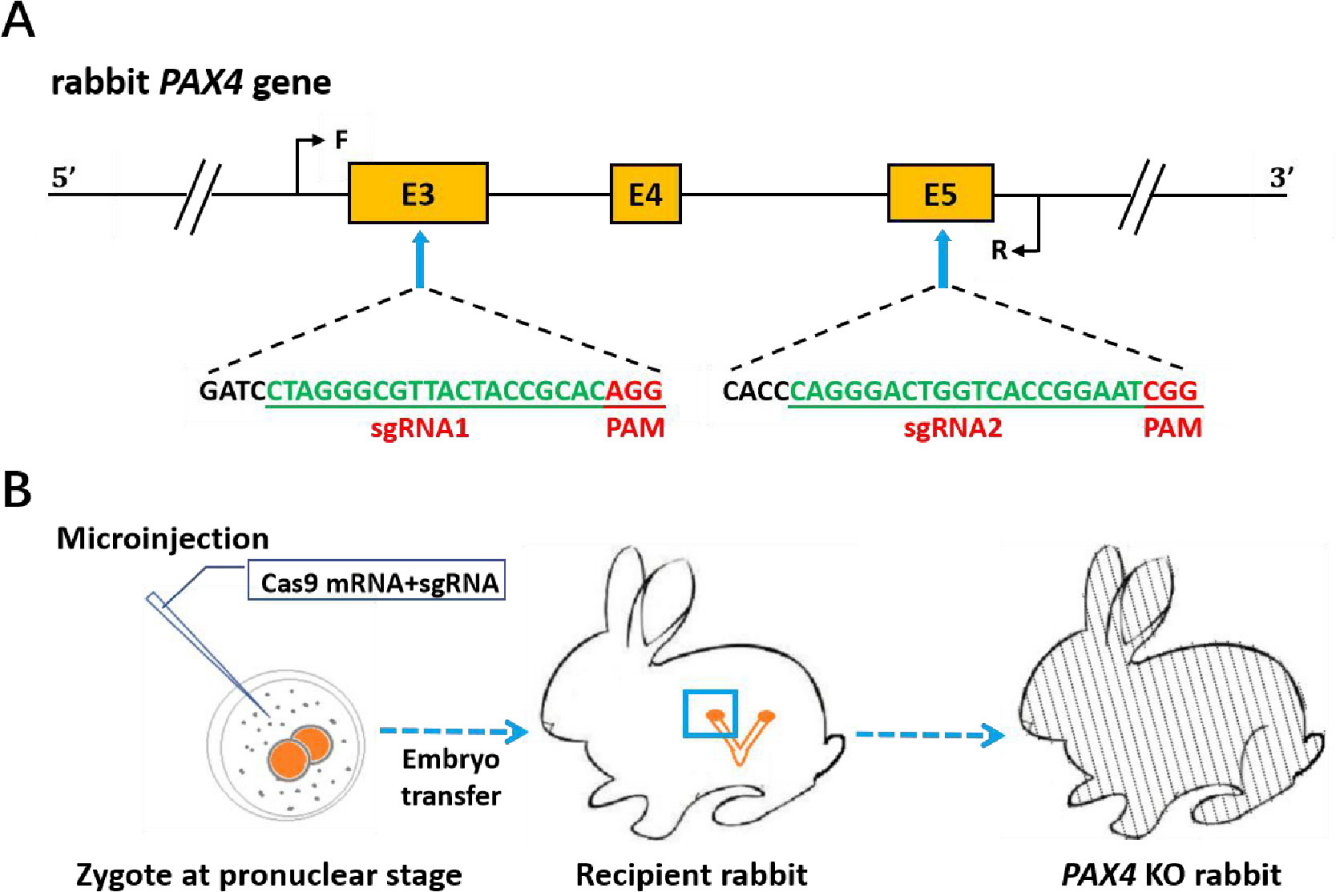
Generation of *PAX4* gene knockout (KO) rabbits using CRISPR-Cas9 system. (A) Schematic diagram of the sgRNA target sites located in the rabbit *PAX4* locus. *PAX4* exons are indicated by yellow rectangles; target sites of the two sgRNA sequences, sgRNA1 and sgRNA2, are highlighted in green; protospacer-adjacent motif (PAM) sequence is highlighted in red. Primers F and R are used for mutation detection in pups. (B) Microinjection and embryo transfer. First a mixture of Cas9 mRNA and sgRNA is microinjected into the cytoplasm of the zygote at the pronuclear stage. Then the injected embryos are transferred into the oviduct of recipient rabbits. After 30 days gestation, *PAX4* KO rabbits are born. (For interpretation of the references to colour in this figure legend, the reader is referred to the web version of this article.)

Our research focuses on the generation of genetically modified rabbit models using CRISPR-Cas systems. Compared with mice, rabbits are closer to humans in physiology, anatomy and evolution [95]. In addition, rabbits have a short gestation period and less breeding cost. All these make them suitable for studies of the cardiovascular, pulmonary and metabolism diseases [95], [96]. Nowadays, we have generated a series of rabbit models for simulating human diseases, including congenital cataracts, duchenne muscular dystrophy (DMD), X-linked hypophosphatemia (XLH), etc (summarized in Table 2) [97], [98], [99], [100], [101], [102], [103], [104], [105], [106], [107], [108], [109], [110], [111], [112], [113], [114]. Take the generation of *PAX4* gene knockout rabbits as an example, the procedure we used to establish genetically modified rabbit models is summarized in Fig. 2 and Table 3.

**Table 2:** CRISPR-Cas system mediated rabbit models of human diseases.

	Rabbit models	Targeted genes	Method	References
1	Congenital Cataracts	CRYAA, Exon 2; GJA8, Exon 1	CRISPR-Cas9, knockout	[97], [98]
2	Muscle hypertrophy	MSTN, Exon 1; MSTN, Exon 1	CRISPR-Cas9, knockout; BE3, point mutation	[99], [113]
3	X-linked hypophosphatemia (XLH)	PHEX, Exon 1	CRISPR-Cas9, knockout	[100]
4	X chromosome inactivation	XIST, D-repeat in Exon 1	CRISPR-Cas9, knockout	[101]
5	Sex reversal	SRY, Sp1	CRISPR-Cas9, knockout	[102]
6	Albinism	Tyr, 3′UTR; Tyr, upstream and 5′UTR (dual sgRNA); Tyr, Exon 1	CRISPR-Cas9, knockout; CRISPR-Cas9, knockout; BE3, point mutation	[103], [104], [113]
7	Diabetes mellitus	PAX4, Exon 3–5	CRISPR-Cas9, knockout	[105]
8	Marfanoid-progeroid-lipodystrophy (MPL) syndrome	FBN1, Exon 65	CRISPR-Cas9, knockout	[106]
9	Pure hair and nail ectodermal dysplasia 9 (ECTD-9)	HOXC13, Exon 1	CRISPR-Cas9, knockout	[107]
10	Duchenne muscular dystrophy (DMD)	DMD, Exon 51	CRISPR-Cas9, knockout	[108]
11	Muscular dystrophy	ANO5, Exon 12–13	CRISPR-Cas9, knockout	[109]
12	Premature Aging Syndrome	LMNA, Exon 3	CRISPR-Cas9, knockout	[110]
13	Autosomal recessive form of hypophosphatemic rickets (ARHR)	DMP1, Exon 1–2	CRISPR-Cas9, knockout	[111]
14	Cleft lip	GADD45G, Exon 2–3	CRISPR-Cas9, knockout	[112]
15	Hutchinson-Gilford progeria syndrome (HGPS)	LMNA, Exon 11	BE3, point mutation	[113]
16	X-linked dilated cardiomyopathy (XLCM)	DMD, Exon 9	ABE7.10, point mutation	[113]
17	Multiple homologous genes knockout	FUT1, FUT2, SEC1, homologous region	CRISPR-Cas9, knockout	[114]

**Table 3:** Summary of the *PAX4* KO rabbits generated by CRISPR-Cas9 system.

Recipients	sgRNA/Cas9 mRNA (ng/μl)	Embryos transferred	Pregnancy	Pups obtained (% transferred)	Pups with mutations (% pups)	Bi-allelic modified (% pups)	Pups with hyperglycemia (% pups)
1	40/200	56	YES	8 (14.2%)	8 (100%)	8 (100%)	8 (100%)
2	40/200	52	YES	6 (11.5%)	6 (100%)	6 (100%)	6 (100%)
3	20/200	52	YES	7 (13.5%)	4 (57.1%)	1 (25%)	1 (25%)
4	20/200	50	YES	1 (2%)	1 (100%)	0	0
Total		210	100%	22 (13.9%)	19 (86.4%)	15 (68.2%)	15 (68.2%)

In addition, the pig is an important model animal extensively used in biomedical research. Compared with mice, their body/organ size, lifespan, anatomy, physiology, metabolic profile and immune characteristics are more similar to those of humans, which makes the pig an ideal model for studying human cardiovascular diseases and xenotransplantation [115]. At present, several genetically modified pig models have been successfully generated, including neurodegenerative diseases, cardiovascular diseases, cancer, immunodeficiency and xenotransplantation model [116], [117], [118], [119], [120], [121], [122].

To date, non-human primates are recognized as the best human disease models. Their advantage is that their genome has 98% homology with the human genome; also, they are highly similar to humans in tissue structure, immunity, physiology and metabolism [123]. What’s more, they can be infected by human specific viruses, which makes them very important models in infectious disease research [124]. Nowadays, researchers have generated many genetically modified monkey models, such as cancer, muscular dystrophy, developmental retardation, adrenal hypoplasia congenita and Oct4-hrGFP knockin monkeys [125], [126], [127], [128], [129].

(2) Establishing cell models of human diseases
  It was found that the efficiency of CRISPR-Cas mediated genome editing is higher *in vitro* than *in vivo*, thus the use of genetically modified cell models can greatly shorten the research time in medical research [130]. Until now, researchers have used CRISPR-Cas systems to perform genetic manipulations on various cell lines, such as tumor cells, adult cells and stem cells, in order to simulate a variety of human diseases [79], [80].

Fuchs *et al.* generated the RPS25-deficient Hela cell line by knocking out ribosomal protein eS25 (RPS25) gene using CRISPR-Cas9 system [131]. Drost *et al.* edited four common colorectal cancer-related genes (APC, P53, KRAS and SMAD4) in human intestinal stem cells (hISCs) by CRISPR-Cas9 technology [132]. The genetically modified hISCs with 4 gene mutations possessed the biological characteristics of intestinal tumors and could simulate the occurrence of human colorectal cancer [132]. Jiang *et al.* induced site-specific chromosome translocation in mouse embryonic stem cells by CRISPR-Cas9, in order to establish a cell and animal model for subsequent research on congenital genetic diseases, infertility, and cancer related to chromosomal translocation [133].

In addition, induced pluripotent stem cells (iPSCs) have shown great application prospect in disease model establishment, drug discovery and patient-specific cellular therapy development [134]. iPSCs have the ability of self-renewal and multiple differentiation potential, which are of great significance in disease model establishment and regenerative medicine research [135]. In recent years, by combining CRISPR-Cas systems with iPSC technology, researchers have generated numerous novel and reliable disease models with isogenic backgrounds and provided new solutions for cell replacement therapy and precise therapy in a variety of human diseases, including neurodegenerative diseases, acquired immunodeficiency syndrome (AIDS), β-thalassemia, etc [134], [135], [136].

### 4.2 Applications of CRISPR-Cas systems in disease diagnosis

With the development of CRISPR-Cas systems and the discovery of novel Cas enzymes (Cas12, Cas13, etc.), CRISPR-based molecular diagnostic technology is rapidly developing and has been selected as one of the world's top ten science and technology advancements in 2018 [137].

Unlike Cas9, Cas13 enzymes possess a ‘collateral cleavage’ activity, which can induce cleavage of nearby non-target RNAs after cleavage of target sequence [54]. Based on the ‘collateral cleavage’ activity of Cas13, Feng Zhang *et al.* [138] developed a Cas13a-based *in vitro* nucleic acid detection platform, named SHERLOCK (Specific High Sensitivity Enzymatic Reporter UnLOCKing). It is composed of Cas13a, sgRNA targeting specific RNA sequences and fluorescent RNA reporters. After Cas13a protein recognizes and cleaves the target RNA, it will cut the report RNA and release the detectable fluorescence signal, so as to achieve the purpose of diagnosis [138]. Researchers have used this method to detect viruses, distinguish pathogenic bacteria, genotype human DNA and identify tumor DNA mutations [137], [138]. Later, Feng Zhang *et al.* improved SHERLOCK system and renamed it as SHERLOCKv2, which can detect four virus at the same time [139].

In addition to Cas13, Cas12 enzymes are also found to possess collateral cleavage activity [140]. Doudna *et al.* [141] developed a nucleic acid detection system based on Cas12a (also known as Cpf1), named DETECTR (DNA endonuclease-targeted CRISPR *trans* reporter)*.* DETECTR has been used to detect cervical cancer associated HPV subtypes (HPV16 and HPV18) in either virus-infected human cell lines or clinical patient samples [141]. Furthermore, Doudna *et al.* are trying to use the newly discovered Cas14 and CasX proteins in molecular diagnosis, which may further enrich the relevant techniques of CRISPR-based molecular diagnosis [142], [143].

CRISPR-based molecular diagnostic technology has incomparable advantages over traditional molecular diagnostic methods, such as high sensitivity and single-base specificity, which is suitable for early screening of cancer, detection of cancer susceptibility genes and pathogenic genes [137], [144]. Meanwhile, CRISPR diagnostics is inexpensive, simple, fast, without special instrument, and is suitable for field quick detection and detection in less-developed areas [137], [144]. At present, many companies are trying to develop CRISPR diagnostic kits for family use, to detect HIV, rabies, Toxoplasma gondi, etc.

### 4.3 Applications of CRISPR-Cas systems in genome-scale screening

CRISPR-Cas9 system enables genome-wide high-throughput screening, making it a powerful tool for functional genomic screening [145]. The high efficiency of genome editing with CRISPR-Cas9 system makes it possible to edit multiple targets in parallel, thus a mixed cell population with gene mutation can be produced, and the relationship between genotypes and phenotypes could be confirmed by these mutant cells [146]. CRISPR-Cas9 library screening can be divided into two categories: positive selection and negative selection [147]. It has been utilized to identify genes associated with cancer cell survival, drug resistance and virus infection in various models [148], [149], [150]. Compared with RNAi-based screening, high-throughput CRISPR-Cas9 library screening has the advantages of higher transfection efficiency, minimal off-target effects and higher data reproducibility [151]. At present, scientists have constructed human and mouse genome-wide sgRNA libraries, and they have been increasingly improved according to different requirements [152], [153]. In the future, CRISPR-Cas9-based high-throughput screening technology will definitely get unprecedented development and application.

### 4.4 Applications of CRISPR-Cas systems in gene therapy

Gene therapy refers to the introduction of foreign genes into target cells to treat specific diseases caused by mutated or defective genes [154]. Target cells of gene therapy are mainly divided into two categories: somatic cells and germ line cells. However, since germ line gene therapy is complicated in technique as well as involves ethical and security issues, today gene therapy is limited to somatic cell gene therapy [155]. Traditional gene therapy is usually carried out by homologous recombination or lentiviral delivery. Nevertheless, the efficiency of homologous recombination is low, and lentiviral vectors are randomly inserted into the recipient genome, which may bring potential security risks to clinical applications [156]. Currently, with the rapid development of CRISPR-Cas systems, they have been widely applied in gene therapy for treating various of human diseases, monogenic diseases, infectious diseases, cancer, etc [155], [156], [157]. Furthermore, some CRISPR-mediated genome-editing therapies have already reached the stage of clinical testing. Table 4 briefly summarizes the ongoing clinical trials of gene therapy using genome-editing technology, including ZFN, TALEN and CRISPR-Cas systems.

(1) Monogenic diseases
  Monogenic diseases refer to the genetic diseases caused by mutations of a single allele or a pair of alleles on a pair of homologous chromosomes [158]. There are more than 6600 known monogenic diseases around the world, β-thalassaemia, sickle cell disease (SCD), hemophilia B (HB), retinitis pigmentosa (RP), leber congenital amaurosis type 10 (LCA10), duchenne muscular dystrophy (DMD), hutchinson-gilford progeria syndrome (HGPS), hereditary tyrosinemia (HT), cystic fibrosis (CF), etc [159]. Most of the monogenic diseases are rare diseases lacking of effective treatment, which will greatly affect the life quality of patients. Nowadays, many animal models of monogenic diseases have been treated with CRISPR-mediated gene therapy. Furthermore, even some CRISPR clinical trials for monogenic diseases are going on [160].

**Table 4:** Summary of clinical trials of gene therapy using genome-editing technology.

Number	Disease	Intervention/treatment	Nuclease	Company/institute	Country	Year	Clinicaltrials.gov ID
1	HIV/HIV Infections	Biological: ZFN modified T cells	ZFN	Sangamo Therapeutics	USA	2009	NCT00842634
2	HIV	Genetic: SB-728mR-HSPC Infusion 3 days following busulfan conditioning	ZFN	Sangamo Therapeutics	USA	2015	NCT02500849
3	HIV	Drug: ZFN Modified CD4 + T Cells	ZFN	National Institute of Allergy and Infectious Diseases (NIAID)	USA	2015	NCT02388594
4	Human Papillomavirus-Related Malignant Neoplasm	Biological: ZFN-603 and ZFN-758	ZFN	Huazhong University of Science and Technology	China	2016	NCT02800369
5	Hemophilia B	Biological: SB-FIX	ZFN	Sangamo Therapeutics	USA	2016	NCT02695160
6	Mucopolysaccharidosis I	Biological: SB-318	ZFN	Sangamo Therapeutics	USA	2016	NCT02702115
7	Mucopolysaccharidosis II	Biological: SB-913	ZFN	Sangamo Therapeutics	USA	2017	NCT03041324
8	HIV	Biological: CD4 CAR^+^CCR5 ZFN T-cells	ZFN	University of Pennsylvania	USA	2018	NCT03617198
9	Transfusion Dependent Beta-thalassemia	Genetic: ST-400 Investigational product	ZFN	Sangamo Therapeutics/	USA	2018	NCT03432364
10	Acute Myeloid Leukemia	Biological: UCART123	TALEN	Cellectis S.A.	USA	2017	NCT03190278
11	Human Papillomavirus-Related Malignant Neoplasm	Biological: TALEN Biological: CRISPR/Cas	TALEN	First Affiliated Hospital, Sun Yat-Sen University	China	2017	NCT03057912
12	Multiple Myeloma	Biological: UCARTCS1A	TALEN	Cellectis S.A.	USA	2019	NCT04142619
13	B-cell Acute Lymphoblastic Leukemia	Biological: UCART22	TALEN	Cellectis S.A.	USA	2019	NCT04150497
14	Acute Myeloid Leukaemia	Biological: UCART123	TALEN	Cellectis S.A	UK	2019	NCT04106076
15	Metastatic Non-small Cell Lung Cancer	Other: PD-1 Knockout T Cells	CRISPR-Cas9	Chengdu MedGenCell, Co., Ltd.	China	2016	NCT02793856
16	HIV-1-infection	Genetic: CCR5 gene modification	CRISPR-Cas9	Affiliated Hospital of Academy to Military Medical Sciences	China	2017	NCT03164135
17	B Cell Leukemia/B Cell Lymphoma	Biological: UCART019	CRISPR-Cas9	Chinese PLA General Hospital	China	2017	NCT03166878
18	EBV positive advanced stage malignancies	PD-1 knockout-T cells from autologous origin	CRISPR-Cas9	The Affiliated Nanjing Drum Tower Hospital of Nanjing University Medical School	China	2017	NCT03044743
19	Esophageal Cancer	Other: PD-1 Knockout T Cells	CRISPR-Cas9	Anhui Kedgene Biotechnology Co.,Ltd	China	2017	NCT03081715
20	T cell malignancy	Genetic: CD7.CAR/28zeta CAR T cells	CRISPR-Cas9	Baylor College of Medicine	USA	2018	NCT03690011
21	Sickle Cell Disease	Biological: CTX001	CRISPR-Cas9	CRISPR Therapeutics	USA	2018	NCT03745287
22	Thalassemia	Biological: iHSCs treatment	CRISPR-Cas9	Allife Medical Science and Technology	USA	2018	NCT03728322
23	β-Thalassemia	Biological: CTX001	CRISPR-Cas9	CRISPR Therapeutics	USA	2018	NCT03655678
24	Solid Tumor	Biological: Mesothelin-directed CAR-T cells	CRISPR-Cas9	Chinese PLA General Hospital	China	2018	NCT03747965
25	B Cell Leukemia/B Cell Lymphoma	Biological: Universal Dual Specificity CD19 and CD20 or CD22 CAR-T Cells	CRISPR-Cas9	Chinese PLA General Hospital	China	2018	NCT03398967
26	Multiple Myeloma/Melanoma/Synovial Sarcoma/Liposarcoma	Biological: NY-ESO-1 redirected autologous T cells with CRISPR edited endogenous TCR and PD-1	CRISPR-Cas9	Parker Institute for Cancer Immunotherapy	USA	2018	NCT03399448
27	Solid Tumor	Biological: anti-mesothelin CAR-T cells	CRISPR-Cas9	Chinese PLA General Hospital	China	2018	NCT03545815
28	Thalassemia Major	Biological: γ-globin reactivated autologous hematopoietic stem cells	CRISPR-Cas9	Shanghai Bioray Laboratory Inc.	China	2019	NCT04211480
29	B-cell malignancies	Biological: CTX110	CRISPR-Cas9	CRISPR Therapeutics AG	USA	2019	NCT04035434
30	β-thalassemia Major	Biological: β-globin restored autologous HSC	CRISPR-Cas9	Shanghai Bioray Laboratory Inc.	China	2019	NCT04205435
31	Leber Congenital Amaurosis 10 (LAC10)	Drug: AGN-151587	CRISPR-Cas9	Editas Medicine, Inc.	USA	2019	NCT03872479
32	CD19^+^ leukemia or lymphoma	Genetic: XYF19 CAR-T cell	CRISPR-Cas9	Xi'An Yufan Biotechnology Co.,Ltd	China	2019	NCT04037566
33	Gastro-Intestinal (GI) Cancer	Biological: Tumor-Infiltrating Lymphocytes (TIL)	CRISPR-Cas9	Intima Bioscience, Inc.	USA	2020	NCT04426669
34	Multiple Myeloma	Biological: CTX120	CRISPR-Cas9	CRISPR Therapeutics AG	USA	2020	NCT04244656
35	Renal Cell Carcinoma	Biological: CTX130	CRISPR-Cas9	CRISPR Therapeutics AG	Australia	2020	NCT04438083
36	Advanced Hepatocellular Carcinoma	Biological: PD-1 knockout engineered T cells	CRISPR-Cas9	Central South University	China	2020	NCT04417764

β-Thalassaemia, a hereditary hemolytic anemia disease, is one of the most common and health-threatening monogenic diseases in the world. It is characterized by mutations in the β-globin (HBB) gene, leading to severe anemia caused by decreased hemoglobin (Hb) level [161]. For the moment, the only way to cure β-thalassemia is hematopoietic stem cell transplantation (HSCT). Yet, high cost of treatment and shortage of donors limit its clinical application [162]. Other therapy, for example, blood transfusion, can only sustain the life of patients but can’t cure the disease [161]. To better treat β-thalassemia, researchers have turned their attention to gene therapy. A major technical idea is to repair the defective β-globin gene of iPSCs from patients with β-thalassemia by CRISPR-Cas9 technology, then red blood cells can be produced normally and the disease could be cured [163], [164]. Besides, reactivating fetal hemoglobin (HbF) expression has also been proposed to be an effective method to treat β-thalassemia through knockout of BCL11A gene, which suppresses the expression of fetal hemoglobin [165], [166].

Additionally, CRISPR-Cas systems have also been used for the treatment of other hematologic diseases, such as sickle cell disease (SCD) and hemophilia B (HB). SCD is a monogenic disease caused by a single-nucleotide mutation in human β-globin gene, leading to a substitution of glutamic acid by valine and the production of an abnormal version of β-globin, which is known as hemoglobin S (HbS) [167]. CRISPR-Cas9 system has been used to treat SCD by repairing the β-globin gene mutation or reactivating HbF expression [168], [169]. HB is an X-linked hereditary bleeding disorder caused by deficiency of coagulation factor IX, and the most common treatment for hemophilia B is supplement blood coagulation factor [170], [171]. Huai *et al.* injected naked Cas9-sgRNA plasmid and donor DNA into the adult mice of F9 mutation HB mouse model for gene correction [172]. Meanwhile, Cas9/sgRNA were also microinjected into germline cells of this HB mouse model for gene correction. Both *in vivo* and *ex vivo* experiment were sufficient to remit the coagulation deficiency [172]. Guan *et al.* corrected the F9 Y371D mutation in HB mice using CRISPR-Cas9 mediated in situ genome editing, which greatly improved the hemostatic efficiency and increased the survival of HB mice [173].

Duchenne muscular dystrophy (DMD) is an X-chromosome recessive hereditary disease, with clinical manifestations of muscle weakness or muscle atrophy due to a progressive deterioration of skeletal muscle function [174]. It is usually caused by mutations in the *DMD* gene, a gene encoding dystrophin protein [174]. Deletions of one or more exons of the *DMD* gene will result in frameshift mutations or premature termination of translation, thereby normal dystrophin protein can not be synthesized [175]. Currently, there is no effective treatment for DMD. Conventional drug treatment can only control the disease to a certain extent, but can not cure it. It was found that a functional truncated dystrophin protein can be obtained by removing the mutated transcripts with CRISPR-Cas9 system [176], [177], [178]. In addition, base editing systems can also be applied in DMD treatment by repairing single base mutation or inducing exon skipping by introducing premature termination codons (PTCs) [179].

Retinitis pigmentosa (RP) is a group of hereditary retinal degenerative diseases characterized by progressive loss of photoreceptor cells and retinal pigment epithelium (RPE) function [180]. RP has obvious genetic heterogeneity, and the inheritance patterns include autosomal dominant, autosomal recessive, and X-linked recessive inheritance [180]. To date, there is still no cure for RP. In recent years, with the rapid development of gene editing technology, there has been some progress in the treatment of RP. Several gene mutations causing RP have been corrected by CRISPR-Cas9 in mouse models to prevent retinal degeneration and improve visual function, for example, RHO gene, PRPF31 gene and RP1 gene [181], [182].

Leber Congenital Amaurosis type 10 (LCA10) is an autosomal retinal dystrophy with severe vision loss at an early age. The most common gene mutation found in patients with LCA10 is IVS26 mutation in the CEP290 gene, which disrupts the coding sequence by generating an aberrant splice site [183]. Ruan *et al.* used CRISPR-Cas9 system to knock out the intronic region of the CEP290 gene and restored normal CEP290 expression [184]. In addition, subretinal injection of EDIT-101 in humanized CEP290 mice showed rapid and sustained CEP290 gene editing [185], [186].

Hutchinson-Gilford Progeria Syndrome (HGPS) is a rare lethal genetic disorder with the characteristic of accelerated aging [187]. A point mutation within exon 11 of lamin A gene activates a cryptic splice site, leading to the production of a truncated lamin A called progerin [188]. However, CRISPR-Cas based gene therapy has opened up a broad prospect in HGPS treatment. Administration of AAV-delivered CRISPR-Cas9 components into HGPS mice can reduce the expression of progerin, thereby improved the health condition and prolonged the lifespan of HGPS mice [189], [190]. In addition, Suzuki *et al*. repaired G609G mutation in a HGPS mouse model via single homology arm donor mediated intron-targeting gene integration (SATI), which ameliorated aging-associated phenotypes and extended the lifespan of HGPS mice [191].

CRISPR-Cas systems have also showed their advantages in gene therapy of hereditary tyrosinemia (HT) and cystic fibrosis (CF). HT is a disorder of tyrosine metabolism caused by deficiency of fuarylacetoacetate hydrolase (Fah) [192]. Yin *et al.* corrected a Fah mutation in a HT mouse model by injecting CRISPR-Cas9 components into the liver of the mice [193]. Then, the wild-type Fah protein in the liver cells began to express and the body weight loss phenotype was rescued [193]. CF, an autosomal recessive inherited disease with severe respiratory problems and infections, has a high mortality rate at an early age [194]. It is caused by mutations in the *CFTR* gene, which encodes an epithelial chloride anion channel, the cystic fibrosis transmembrane conductance regulator (CFTR) [194]. Until now, genome editing strategies have been carried out in cell models to correct CFTR mutations. In cultured intestinal stem cells and induced pluripotent stem cells from cystic fbrosis patients, the *CFTR* homozygous Δ508 mutation has been corrected by CRISPR-Cas9 technology, leading to recovery of normal CFTR expression and function in differentiated mature airway epithelial cells and intestinal organoids [195], [196].

(2) Infectious diseases
  In recent years, gene therapy has gradually been applied to the treatment of viral infectious diseases. Transforming host cells to avoid viral infection or preventing viral proliferation and transmission are two main strategies for gene therapy of viral infectious diseases [197].

Human immunodeficiency virus (HIV), a kind of retrovirus, mainly attacks the human immune system, especially the CD4^＋^ T lymphocytes. When human cells are invaded by HIV, the viral sequences can be integrated into the host genome, blocking cellular and humoral immunity while causing acquired immunodeficiency syndrome (AIDS) [198]. There is still no known cure for AIDS but it could be treated. Although antiretroviral therapy can inhibit HIV-1 replication, the viral sequences still exist in the host genome, and they could be reactivated at any time [199]. CRISPR-Cas9 system can target long terminal repeat (LTR) and destruct HIV-1 proviruses, thus it is possible to completely eliminate HIV-1 from genome of infected host cells [200], [201]. In addition, resistance to HIV-1 infection could be induced by knockout of the HIV co-receptor CCR5 gene in CD4^＋^ T cells [202], [203].

Cervical cancer is the second most common gynecologic malignant tumor. The incidence is increasing year by year and young people are especially prone to this disease. It was found that the occurrence of cervical cancer is closely related to HPV (human papillomavirus) infection [204]. HPV is a double-stranded cyclic DNA virus, E6 and E7 genes located in HPV16 early regions are carcinogenic genes [205]. Researchers designed sgRNAs targeting E6 and E7 genes to block the expression of E6 and E7 protein, subsequently the expression of p53 and pRb was restored to normal, finally increasing tumor cells apoptosis and suppressing subcutaneous tumor growth in *in vivo* experiments [206], [207], [208]. Moreover, HPV virus proliferation was blocked through cutting off E6/E7 genes, and the virus in the bodies could be eliminated [206], [207], [208].

(3) Cancer
  Cancer is the second leading cause of death worldwide after cardiovascular diseases, and it is also a medical problem that needs to be solved urgently. A variety of genetic or epigenetic mutations have been accumulated in the cancer genome, which can activate proto-oncogenes, inactivate tumor suppressors and produce drug resistance [209], [210]. So far, CRISPR-Cas systems have been used to correct the oncogenic genome/epigenome mutations in tumor cells and animal models, resulting in inhibition of tumor cell growth and promotion of cell apoptosis, thereby inhibiting tumor growth [211], [212], [213].

In addition, immunotherapy is considered to be a major breakthrough in cancer treatment, especially chimeric antigen receptor-T (CAR-T) cell therapy, which has a significantly therapeutic effect on leukemia, lymphoma and certain types of solid tumors [214], [215], [216]. CAR-T cells are genetically manipulated, patient-specific T cells, which express receptors targeting antigens specially expressed on tumor cells, for example, CD19 CAR-T cells for B cell malignancies. Then these cells will be transfused back to patients to fight against cancer [217]. However, CAR-T cell therapy is complex, time-consuming and expensive, and it is greatly limited by the quality and quantity of autologous T cells. Therefore, researchers have used CRISPR-Cas9 system to develop universal CAR-T cells, such as simultaneously removing endogenous T cell receptor gene and HLA class I encoding gene on T cells of healthy donors and introducing CAR sequence [218], [219], [220]. Thereby, it could be used in multiple patients without causing graft versus host reaction (GVHR). In addition, CRISPR-Cas mediated genome editing has also been used to enhance the function of CAR-T cells by knocking out genes encoding signaling molecules or T cell inhibitory receptors, such as programmed cell death protein 1 (PD-1) and cytotoxic T lymphocyte antigen 4 (CTLA-4) [221], [222].

## 5 Challenges and perspectives

Though CRISPR-Cas mediated efficient genome editing technologies have been broadly applied in a variety of species and different types of cells, there are still some important issues needed to be addressed during the process of application, such as off-target effects, delivery methods, immunogenicity and potential risk of cancer.

### 5.1 Off-target effects

It was found that designed sgRNAs will mismatch with non-target DNA sequences and introduce unexpected gene mutations, called off-target effects [223]. Off-target effects seriously restrict the widespread application of CRISPR-Cas mediated genome editing in gene therapy, for it might lead to genomic instability and increase the risk of certain diseases by introducing unwanted mutations at off-target sites [224]. At present, several strategies have been used to predict and detect off-target effects, online prediction software, whole genome sequencing (WGS), genome-wide, unbiased identification of DSBs enabled by sequencing (GUIDE-seq), discovery of in situ cas off-targets and verification by sequencing (DISCOVER-Seq), etc [225]. Furthermore, to minimize off-target effects, researchers have systematically studied the factors affecting off-target effects and developed a number of effective approaches.

(1) Rational design and modification of sgRNAs
  The specific binding of sgRNA with the target sequence is the key factor in CRISPR-Cas mediated genome editing. Rational design of highly specific sgRNAs might minimize off-target effects [224]. The length and GC content of sgRNAs, and mismatches between sgRNA and its off-target site will all affect the frequency of off-target effects [226]. In addition, on the basis of rational design of sgRNAs, the specificity of CRISPR-Cas systems can be further improved by modifying sgRNAs, such as engineered hairpin sgRNAs and chemical modifications of sgRNAs [227], [228].
(2) Modification of Cas9 protein
  As we know, the interaction between Cas9 and DNA affects the stability of DNA-Cas9/sgRNA complex as well as tolerance to mismatch [229]. Therefore, high-fidelity SpCas9 variants have been developed by introducing amino substitution(s) into Cas9 protein in order to destabilize the function structure of the CRISPR complex [230]. Researchers have developed several highly effective Cas9 mutants, high-fidelity Cas9 (SpCas9-HF1), enhanced specificity Cas9 (eSpCas9), hyper-accurate Cas9 (HypaCas9), etc [231], [232], [233]. All of them can significantly reduce off-target effects while retain robust target cleavage activity.
(3) Adoption of double nicking strategy
  Recently, a double-nicking strategy has been developed to minimize off-target effects, which employs two catalytic mutant Cas9-D10A nickases and a pair of sgRNAs to produce a cleavage on each strand of the target DNA, thus forming a functional double strand break [234]. Additionally, it was proven that the fusion protein generated by combining dCas9 with *Fok*Ⅰ nuclease can also reduce off-target effects [235]. Only when the two fusion protein monomers are close to each other to form dimers, can they perform the cleavage function [235]. This strategy could greatly reduce DNA cleavage at non-target sites.
(4) Anti-CRISPRs
  “Off switches” for CRISPR-Cas9 system was first discovered by Pawluk *et al.* in 2016. They identified three naturally existing protein families, named as “anti-CRISPRs”, which can specifically inhibit the CRISPR-Cas9 system of *Neisseria meningitidis* [236]. Later, Rauch *et al.* discovered four unique type IIA CRISPR-Cas9 inhibitor proteins encoded by *Listeria monocytogenes* prophages, and two of them (AcrllA2 and AcrllA4) can block SpCas9 when assayed in *Escherichia coli* and human cells [237]. Recently, Doudna *et al.* discovered two broad-spectrum inhibitors of CRISPR-Cas9 system (AcrllC1 and AcrllC3) [238]. Therefore, in order to reduce off-target effects, the “anti-CRISPRs” could be used to prevent the continuous expression of Cas9 protein in cells to be edited.
(5) Others
  The concentration of Cas9/sgRNA can also affect the frequency of off-target mutations [239]. Thus, the optimal concentration of Cas9 and sgRNA needs to be determined by pre-experiment. Besides, the formulation of CRISPR-Cas9 can affect the frequency of off-target mutations as well. Cas9 nucleases can be delivered into target cells in 3 different forms: DNA expression plasmid, mRNA or recombination protein [240]. Currently, the use of Cas9/sgRNA ribonucleoprotein complexes (Cas9-RNPs), which are composed of purified Cas9 proteins in combination with sgRNA, is becoming more and more widespread. It was found that delivery as plasmid usually produces more off-targets than delivery as RNPs, since the CRISPR-Cas system is active for a shorter time without Cas9 transcription and translation stages [241], [242].

### 5.2 Delivery methods

Nowadays, how to effectively deliver CRISPR-Cas components to specific cells, tissues and organs for precisely directed genome editing is still a major problem in gene therapy. Ideal delivery vectors should have the advantages of non-toxicity, well targeting property, high efficiency, low cost, and biodegradability [35], [156]. At present, three main delivery methods have been employed in delivering CRISPR-Cas components, including physical, viral and non-viral methods [243]. Physical methods are the simplest way to deliver CRISPR-Cas components, including electroporation, microinjection and mechanical cell deformation. They are simple and efficient, which can also improve the expression of genes, and being widely applied in *in vitro* experiments [243], [244]. In addition, viral vectors, such as adenovirus, adeno-associated virus (AAV) and lentivirus viral vectors, are being widely used for both *in vitro*/*ex vivo* and *in vivo* delivery due to their high delivery efficiency. They are commonly used for gene delivery in gene therapy, and some of them have been approved for clinical use [245], [246]. However, safety issue of viral vectors is still a major problem needed to be solved in pre-clinical trials. Therefore, researchers have turned their attention to non-viral vectors, for instance, liposomes, polymers and nanoparticles [247]. Based on the advantages of safety, availability and cost-effectiveness, they are becoming a hotspot for the delivery of CRISPR-Cas components [248].

Since all these delivery methods have both advantages and disadvantages, it’s necessary to design a complex of viral vectors and non-viral vectors, which combines the advantages of both vectors. Along with the deepening of research, various carriers could be modified by different methods to increase the delivery efficiency and reduce the toxicity [249]. In addition, more novel vectors, such as graphene and carbon nanomaterials (CNMs), could also be applied in the delivery of CRISPR-Cas components [250], [251].

### 5.3 Immunogenicity

Since the components of CRISPR-Cas systems are derived from bacteria, host immune response to Cas gene and Cas protein is regarded as one of the most important challenges in the clinical trials of CRISPR-Cas system [156], [252]. It was found that *in vivo* delivery of CRISPR-Cas components can elicit immune responses against the Cas protein [252], [253]. Furthermore, researchers also found that there were anti-Cas9 antibodies and anti-Cas9 T cells existing in healthy humans, suggesting the pre-existing of humoral and celluar immune responses to Cas9 protein in humans [254]. Therefore, how to detect and reduce the immunogenicity of Cas proteins is a major challenge will be faced in clinical application of CRISPR-Cas systems. Researchers are trying to handle this problem by modifying Cas9 protein or using Cas9 homologues [255].

### 5.4 Potential risk of cancer

Recently, two independent research groups found that CRISPR-Cas mediated double-stranded breaks (DSBs) can activate the p53 signaling pathway [256], [257]. This means that genetically edited cells are likely to become potential cancer initiating cells, and clinical treatment with CRISPR-Cas systems might inadvertently increase the risk of cancer [256], [257], [258]. Although there is still no direct evidence to confirm the relationship between CRISPR-Cas mediated genome editing and carcinogenesis, these studies once again give a warning on the application of CRISPR-Cas systems in gene therapy. It reminds us that there is still a long way to go before CRISPR-Cas systems could be successfully applied to humans.

### 5.5 Ethical issues

CRISPR-Cas mediated genome editing has attracted much attention since its advent in 2012. In theory, each gene can be edited by CRISPR-Cas systems, even genes in human germ cells [259]. However, germline gene editing is forbidden in many countries including China, for it could have unintended consequences and bring ethical and safety concerns [260].

However, in March 2015, a Chinese scientist, Junjiu Huang, published a paper about gene editing in human tripronuclear zygotes in the journal Protein & Cell, which brings the ethical controversy of human embryo gene editing to a climax [261]. Since then, genome editing has been challenged by ethics and morality, and legal regulation of genome editing has triggered a heated discussion all around the world.

Then, on Nov. 28, 2018, the day before the opening of the second international human genome editing summit, Jiankui He, a Chinese scientist from the Southern University of Science and Technology, announced that a pair of gene-edited babies, named Lulu and Nana, were born healthy in China this month. They are the world’s first gene-edited babies, whose CCR5 gene has been modified, making them naturally resistant to HIV infection after birth [262]. The announcement has provoked shock, even outrage among scientists around the world, causing widespread controversy in the application of genome editing.

The society was shocked by this breaking news, for it involves genome editing in human embryos and propagating into future generations, triggering a chorus of criticism from the scientific community and bringing concerns about ethics and security in the use of genome editing. Therefore, scientists call on Chinese government to investigate the matter fully and establish strict regulations on human genome editing. Global supervisory system is also needed to ensure genome editing of human embryos moving ahead safely and ethically [263].

### 5.6 Conclusions

Since CRISPR-Cas mediated genome editing technologies have provided an accessible and adaptable means to alter, regulate, and visualize genomes, they are thought to be a major milestone for molecular biology in the 21st century. So far, CRISPR-Cas systems have been broadly applied in gene function analysis, human gene therapy, targeted drug development, animal model construction and livestock breeding, which fully prove their great potential for further development. However, there are still some limitations to overcome in the practical applications of CRISPR-Cas systems, and great efforts still need to be made to evaluate their long-term safety and effectiveness.

## CRediT authorship contribution statement

**Yuanyuan Xu:** Conceptualization, Writing - original draft. **Zhanjun Li:** Supervision, Validation, Writing - review & editing.

## Declaration of Competing Interest

The authors declare that they have no known competing financial interests or personal relationships that could have appeared to influence the work reported in this paper.
